# A Fast and Highly Selective Nitrite Sensor Based on Interdigital Electrodes Modified With Nanogold Film and Chrome-Black T

**DOI:** 10.3389/fchem.2020.00366

**Published:** 2020-04-29

**Authors:** Haoyue Luo, Xiaogang Lin, Zhijia Peng, Yong Zhou, Shibin Xu, Ming Song, Lifeng Jin, Xiaodong Zheng

**Affiliations:** ^1^Key Laboratory of Optoelectronic Technology and Systems of Ministry of Education of China, Chongqing University, Chongqing, China; ^2^Chongqing University Cancer Hospital, Chongqing University, Chongqing, China

**Keywords:** interdigital electrode sensors, nanogold film, detection of nitrite, impedance, capacitance

## Abstract

Nitrite is a toxic substance, when excessive nitrite enters the human body, it will be seriously harmful to human. At present, the detection methods of nitrite are complicated to operate and require expensive detection instruments. Therefore, an effective, fast and highly selective nanogold film interdigital electrode sensors that can detect nitrite easily and quickly is developed in the work. Firstly, the variation of the sensitivity of nanogold film nitrite sensors with concentrations (1 mol/L, 10^−1^ mol/L, 10^−2^ mol/L, 10^−3^ mol/L, 10^−4^ mol/L, and 10^−5^ mol/L) was measured by experiments. Then, Chrome-black T was modified to the surface of the nanogold film interdigital electrodes by electrochemical polymerization, and the film of chrome-black T had affinity for nitrite ions, so nitrite ions were enriched on the sensor surface. The change law of the impedance signal of the modified nanogold film nitrite sensors after being added to different concentrations of sodium nitrite solution were also concluded. The study demonstrates that the larger the concentration of sodium nitrite solution is added to the modified interdigital electrodes, the smaller impedance and resistance of the modified interdigital electrodes are reflected. Finally, specificity of the modified interdigital electrode sensors has been demonstrated. The novel interdigital electrode sensors can detect the concentration of nitrite solution conveniently and quickly with only 30 s. Therefore, the prospect of applying the novel nanogold film interdigital electrode sensors to the detection of nitrite in blood, body fluid, food and drinking water is promising.

## Introduction

Nitrite is a food preservative and therefore commonly used as a food preservative. So humans are ubiquitously exposed to nitrite. It will destroy the function of red blood cells when excessive nitrite enters the human body. What's worse, it may result in death. In addition, Nitrite is also able to react with other compounds to produce carcinogens under specific conditions.

A highly sensitive, simple, and rapid method to detect nitrite in foods and water is essential for us. At present, there are several methods to detect nitrite. The detection of nitrite by spectroscopic method is one of the most basic and common methods, but it is easy to be interfered by impurities with low accuracy and sensitivity. A surface-enhanced Raman spectroscopy (SERS) method coupled with a commercially available gold nano substrate was evaluated for the detection of nitrate and nitrite in water by Gajaraj et al. (2013). Chromatographic method has the advantages of simple operation and wide linear range. But toxic agents and the expensive instruments should be used in the detection, which has impeded its development. Akio Tanaka used an electron capture detector to analyze nitrite in meat and cheese by gas-liquid chromatography (Tanaka et al., 1982). A method was developed for determination of inorganic anions, including nitrite (NO_2_-) and nitrate (NO_3_-) in seawater by ion chromatography (Ito et al., 2012). In recent years, electrochemical sensors are widely used to measure the content of nitrite in water and food. It has the advantages of easy operation, high sensitivity and good stability. The development of an electrode system for the determination of nitrite was presented. The approach was based upon the deposition of a macroporous copper deposit which showed marked specificity for nitrite ion under mildly acidic conditions (pH 3) with a linear range extending from 10 to 200 μmol/L nitrate (Davis et al., 2000). Xu et al. used a glassy carbon electrode modified by combining bovine hemoglobin with nanometer gold-reduced graphene oxide to detect nitrite, they found that the modified electrode had good specificity and sensitivity, and had wide linear range of detection: 0.5–100 μmol/L, the minimum detection limit of 0.1 μmol/L (Xu et al., 2015). A paper test-strip technology, used in conjunction with a modern hand-held reflectometer was tested to permit fine spatial and temporal resolution analysis of nitrite breakthrough in a large undisturbed soil block. The techniques proved cost effective and had the added benefit of stopping locally generated toxic waste (Holden and Scholefield, 2008; Jiang et al., 2018). However, some of these methods have low accuracy and sensitivity, some of them are time-consuming and require expensive apparatuses which are also operated by highly expert experimenter.

In order to develop a highly sensitive, simple, and rapid method, we used nanogold film interdigital electrode sensors to detect nitrite in this study. The advantages of the nanogold film interdigital electrode sensors are convenient, economical, and high throughput capacity. Purified water was used as the background solution in experiments. We detected the frequency sweep curve and the alternating voltage sweep curve of the nanogold film interdigital electrode sensors to find the changes of their impedance. But the nanogold film interdigital electrode sensors didn't own good specificity and stability. Therefore, Chrome-black T was modified to the surface of the interdigitated electrode by electrochemical polymerization, and the film of chrome-black T have affinity for nitrite ions. The change law of the impedance, capacitance, and resistance signals of the modified nanogold film nitrite sensors were further concluded. Finally, we have also demonstrated the specificity of the modified interdigital electrode sensors in the work.

## Materials and Methods

### Materials

The sodium nitrite was purchased from Tansoole (China). The sodium nitrite was firstly dissolved in purified water to make a 1 mol/L stock solution and stored at room temperature. Then the stock solution was further diluted in purified water to make the working solution at 10^−1^ mol/L, 10^−2^ mol/L, 10^−3^ mol/L, 10^−4^ mol/L, and 10^−5^ mol/L for testing. At the same time, the purified water was used as the background solution. Chrome-black T and Sodium hydroxide were also purchased from Tansoole (China). Chrome-black T and Sodium hydroxide were dissolved in purified water to make the solution which included 1 mmol/L Chrome-black T and 10 mmol/L Sodium hydroxide for modifying. All other reagents were of analytical grade (Lin et al., 2019a,b). At the same time, the sodium nitrite mixture solution was also prepared with the concentration of 10^−1^ mol/L, in which the concentration of carbonate ion was 0.1 mol/L, the concentration of chloride ion was 0.1 mol/L, the concentration of nitrate ion was 0.1 mol/L, the concentration of magnesium ion was 0.05 mol/L, and the concentration of potassium ion was 0.3 mol/L. Then the sodium nitrite mixture solution was further diluted in purified water to make the sodium nitrite mixture solution at 10^−1^ mol/L, 10^−2^ mol/L, 10^−3^ mol/L, 10^−4^ mol/L, and 10^−5^ mol/L for specificity testing. And the mixture solution without the sodium nitrite was also prepared for testing.

### Detection Mechanisms

**Figure 1:**
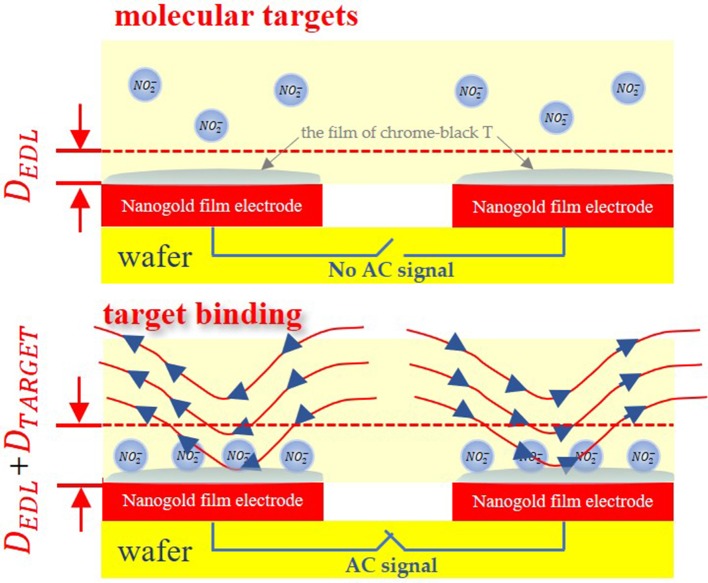
Mechanism of capacitive sensing.

In this study, we utilized nanogold film interdigital electrodes as the sensors. The interdigital microelectrodes are finger-shaped in its surface. At the same time, the tiny size of the interdigital microelectrodes is able to achieve microminiaturization. When nanogold film interdigital microelectrodes are immersed in solution, the electrodes impedance can be approximated as a network of resistors and capacitors, as conceptually shown in Figure 1. Electrical double layer (EDL) is a structure that appears on the surface of nanogold film when it is exposed to a fluid. And EDL can be modeled as a capacitor. When sodium nitrite molecules are adsorbed onto the nanogold film surface, the interfacial capacitance *C*_*int*_ will change due to the change in the thickness and surface area of *C*_*int*_, which can then be used to indicate the deposition of sodium nitrite molecules on the nanogold film (Li et al., 2015; Lin et al., 2017).

Before the affinity assay, the electrode surface is immobilized with the film of chrome-black T to achieve selectivity for the targeted particles. The interfacial capacitance can be approximated by following equation.

(1) $$\begin{array}{l} C_{int}=\frac{A}{\frac{1}{\varepsilon_{s}}D_{edl}+\frac{1}{p}D_{target}} \end{array}$$

Where ε_*s*_ and ε_*p*_ are permittivity of the sample and target molecules, A is the surface area and *D*_*edl*_, *D*_*target*_ are electric double layer, target thickness respectively.

What's more, when AC signals are applied to the interdigital electrodes, an AC electric field is able to generate microflows through AC electroosmosis (Wu et al., 2005; Mirzajani et al., 2016) or AC electrothermal (Hart et al., 2010) effect. And these mechanisms can accelerate detection. This method has been used to detect small molecules, proteins, virus particles, and nuclei acids (Cui et al., 2015; Liu et al., 2016; Cheng et al., 2017a,b). In this work, the sample solutions were added to the interdigital electrodes, which could alter electrodes' impedance, so the law of changes of the impedance was used to demonstrate the concentration of sample solution.

### Preparation of Nanogold Film Interdigital Microelectrodes

In this work, the nanogold film interdigital microelectrodes were fabricated on silicon wafers. And there were four kinds of microelectrodes. We test all of them in order to select the optimal one. At last, we used the nanogold film interdigital microelectrodes which had interdigitated arrays with widths of 10 μm separated by 10 μm gaps to accomplish the test work.

**Figure 2:**
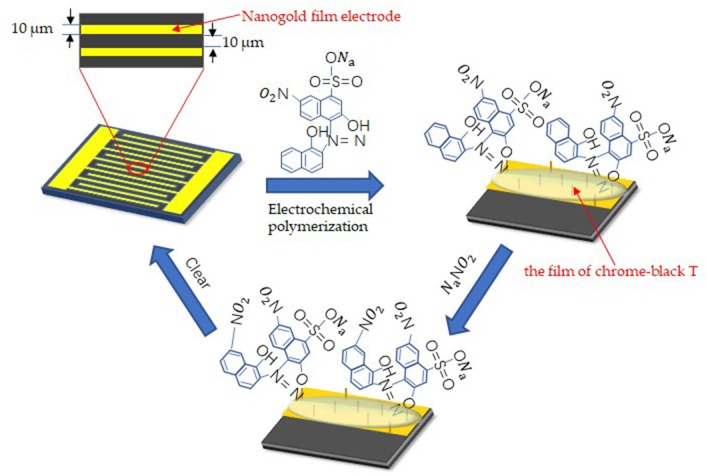
Surface modification techniques on the interdigital electrodes surface for the detection of sodium nitrite.

Before the detection, the nanogold film interdigital microelectrodes should be cleaned with the following steps: immersed in acetone for 4 min with ultrasonic cleaning; rinsed in absolute ethyl alcohol for 3 min with ultrasonic cleaning; rinsed in deionized water for 3 min with ultrasonic cleaning; dried with drying oven. After pretreatment, Chrome-black T was modified to the surface of the electrode by electrochemical polymerization. In order to form the film of chrome-black T, 10 μL of solution which included 1 mmol/L Chrome-black T and 10 mmol/L Sodium hydroxide was added to the nanogold film interdigital microelectrodes. At the same time, alternating voltage that differed from 0.01 to 0.9 V were applied to the microelectrodes with a settled frequency (10 kHz). And the number of times of alternating voltage sweep were 25. Figure 2 shows the process of surface modification on the nanogold film interdigital microelectrodes and the affinity for nitrite ions. Here, we get the nanogold film nitrite sensors.

### Apparatus and Methods

Firstly, an impedance analyzer of model IM3536 (HIOKI, Japan) was used to obtain the impedance datum of the bare nanogold film interdigital electrodes with different concentrations of sodium nitrite solutions. In the first stage, the frequency that differed from 100 Hz to 100 kHz were applied to the microelectrodes when they were under a settled alternating voltage. In the fact, we conducted frequency scanning on the bare interdigital microelectrodes. At the same time, we set the alternating voltage as nine equal voltage gradients which differed from 10 to 90 mv. Ten microliter of different concentrations of sodium nitrite solutions were added to the bare nanogold film interdigital microelectrodes. Then the impedance of the bare interdigital microelectrodes was detected. In the second stage, continuous alternating voltage which differed from 10 to 90 mv were applied to the bare interdigital microelectrodes when they were under a fixed frequency. In the same way, the impedance of the bare interdigital microelectrodes was detected when 10 μL of different concentrations of sodium nitrite solutions were added to them.

The impedance of nanogold film interdigital microelectrodes was reflected in the frequency sweep curve when different concentrations of sodium nitrite solution were added to them. Furthermore, the impedance of bare interdigital microelectrodes was also reflected in the alternating voltage sweep curve when different concentrations of sodium nitrite solution were added to them.

Secondly, in order to demonstrate the specificity of the modified nanogold film interdigital electrode sensors, the impedance analyzer of model IM3536 (HIOKI, Japan) was used to obtain the impedance, capacitance, resistance signals of the modified nanogold film nitrite sensors. A 10 kHz AC signal of 10 mV was used for pumping signal. And the voltage used in the experiments is rms. Ten microliter of different concentrations of working solutions were added to the sensors. Then the impedance, capacitance and resistance signals of the modified nanogold film interdigital electrodes were continuously measured at the AC signal for 30 s. We tested three times for each experimental datum. And we used the average of the experimental datum to analyze (Tang et al., 2019). The change rate of normalized capacitance was then calculated to indicate the binding level of sodium nitrite, which is shown as the slope of normalized capacitance vs. time (%/min), found by a least square linear fitting method. The normalized capacitance was calculated as *C*_*t*_*/C*_0_, where *C*_*t*_ and *C*_0_ are the capacitance values at time *t* and time zero, respectively. The capacitance of each biosensor may be quite different. But the normalized capacitance can reduce the errors caused by the difference of each biosensor. So it can reduce the influence on the results.

## Results and Discussion

### Detection in Fixed Alternating Voltage

**Figure 3:**
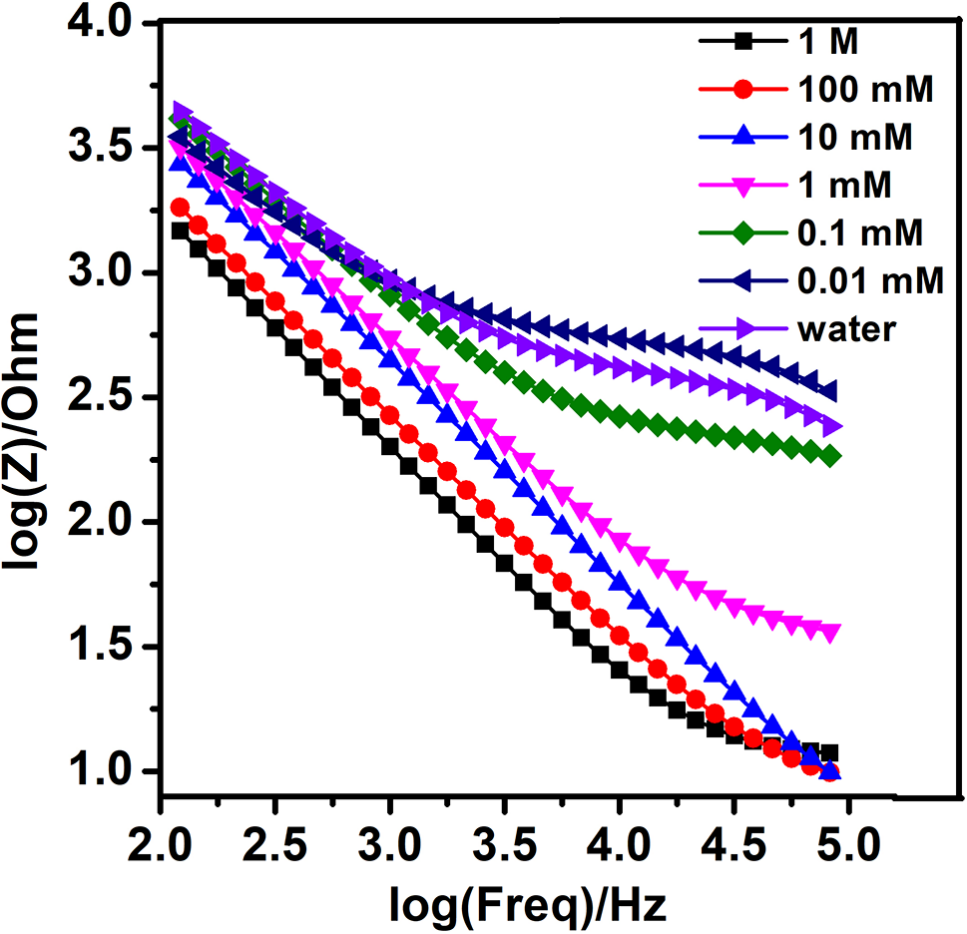
Bode diagram of different concentrations of nitrite with AC signal of 10 mV.

In the first stage, in order to evaluate the performance of the bare nanogold film interdigital electrodes, the optimal interdigital electrode (10 μm widths, 10 μm gaps) was tested with different concentrations of the sodium nitrite solutions in fixed alternating voltages (Zhou et al., 2019). And the frequency that differed from 100 Hz to 100 kHz and a fixed alternating voltage were set well. The detections were repeated three times. At last, 10 mV was selected from various fixed alternating voltages. At the fixed alternating voltage, the Bode diagram demonstrated the relationship between concentrations of the sodium nitrite solutions and the impedance of interdigital microelectrodes which were added to these working solutions. As is shown in Figure 3, the larger concentration of sodium nitrite solution is added to the bare nanogold film interdigital electrodes, the faster reduction rate of the bare nanogold film interdigital electrodes' impedance presents. At the same time, the change rate of the sodium nitrite solution at 10^−5^ mol/L is approximately consistent with that of purified water.

### Detection in Fixed Frequency

**Figure 4:**
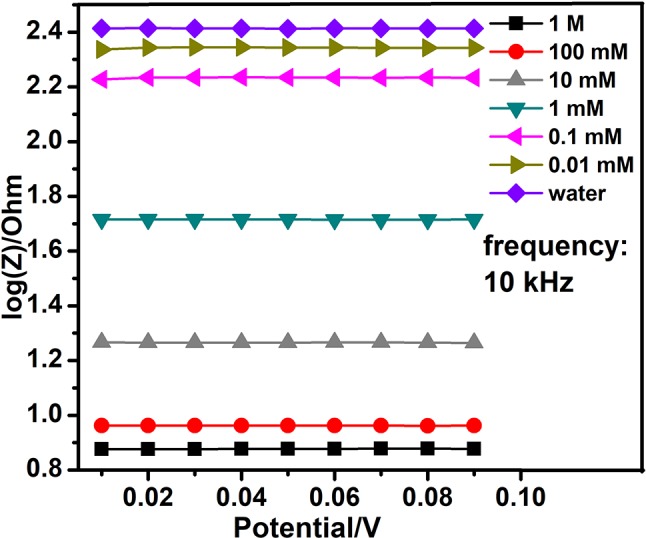
The voltage sweep of different concentrations of nitrite with AC signal of 10 kHz.

On the base of the previous work, we tested the optimal interdigital electrode again with different concentrations of the sodium nitrite solutions in fixed frequencies. In the second stage, the alternating voltage that differed from 10 to 90 mv and a fixed frequency were set well. The detections were repeated three times. At last, 10 kHz was selected from various fixed frequencies. At the fixed frequency, the alternating voltage sweep curve demonstrated the relationship between concentrations of the sodium nitrite solutions and the impedance of interdigital microelectrodes which were added to these working solutions. The Figure 4 evidently shows that the larger concentration of sodium nitrite solution is added to the bare nanogold film interdigital electrodes, the smaller impedance of the bare nanogold film interdigital electrodes presents.

### Specificity Studies of the Modified Interdigital Electrode Sensors

**Figure 5:**
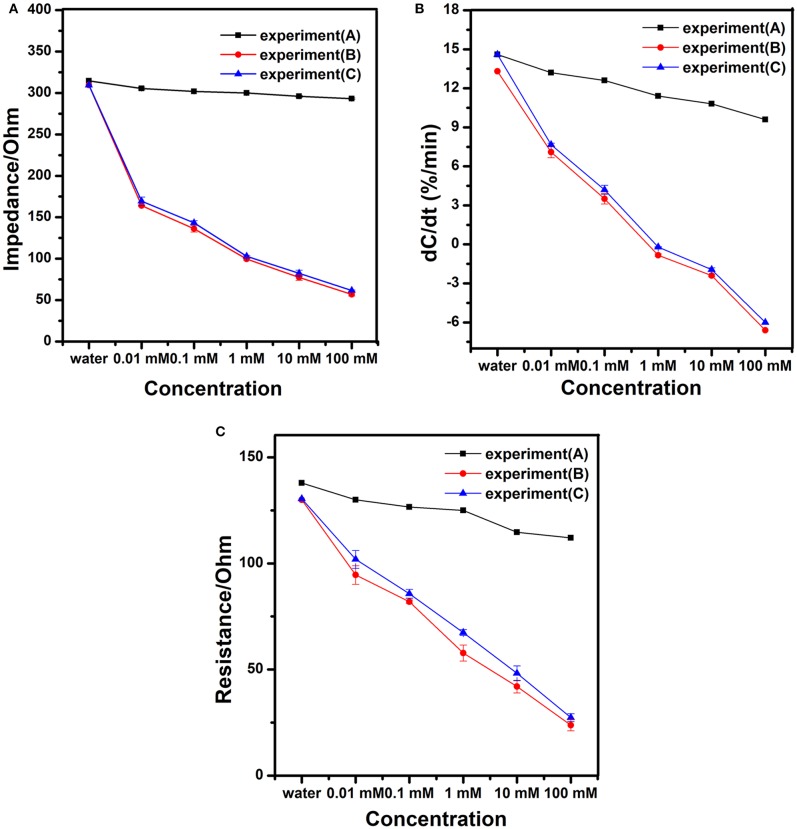
Diagrams of **(A)** the impedance, **(B)** the change rate of normalized capacitance, and **(C)** the resistance of the modified interdigital electrode sensor after being added different concentrations of working solutions.

Different concentrations of working solutions were tested to evaluate the performance of the modified nanogold film interdigital electrode sensors. Based on the 10 kHz and 10 mV AC excitation signal, we measured the impedance of the modified nanogold film interdigital electrode sensors for 30 s. Three experiments were designed to verify the specificity of the sensor. The impedance of different concentrations of mixture solutions without sodium nitrite were measured in experiment (A). The impedance of different concentrations of sodium nitrite solutions were measured in experiment (B). The impedance of different concentrations of sodium nitrite mixture solutions were measured in experiment (C). The sodium nitrite mixture solution includes nitrite ion, carbonate ion, chloride ion, nitrate ion, magnesium ion and potassium ion. As is shown in Figure 5A, the larger concentration of sodium nitrite solution is added on the sensors, the smaller impedance of the sensors present. From Figure 5B, we can see a correlation between concentrations of sodium nitrite solutions and the change rate of normalized capacitance. And Figure 5C shows that the law of changes of the modified nanogold film interdigital electrode sensor's resistance is similar to the impedance after being added different concentrations of sodium nitrite solutions. The impedance, capacitance, and resistance are common electrical parameters of biosensor for detecting. At the same time, Figure 5 shows an excellent specificity of the modified nanogold film interdigital electrode sensors. When different concentrations of sodium nitrite mixture solutions were tested, there was nearly no effect on the detection of the nitrite ions. While when different concentrations of mixture solutions without sodium nitrite were tested, the result was similar to that of purified water. Because of the AC electrokinetic effect, some other ions in the mixture solutions may be accelerated to deposit on the surface of the electrode. So the value of impedance, normalized capacitance and resistance decrease slightly when different concentrations of mixture solutions without sodium nitrite were added to the modified interdigital electrode sensors. However, the decreases don't have influences on the results. What's more, the results between mixture solutions without sodium nitrite and sodium nitrite solutions had obvious difference in Figure 5.

## Conclusions

In this research, we developed an effective, rapid, and high specificity method for detection of nitrite in water. The larger concentration of sodium nitrite solution is dropped on the modified nanogold film interdigital electrode sensors, the smaller impedance of the modified nanogold film interdigital electrode sensors has. In this work, we selected the optimal dimension of the interdigital electrode sensors and the best experiment conditions for detection. The developed modified nanogold film interdigital electrode sensors have the high specificity to the nitrite ions when different concentrations of sodium nitrite mixture solutions were tested. And the nitrite biosensor can detect the concentration of nitrite solution quickly with only 30 s. At the same time, the limit of detection (LOD) of the developed sensors is nearly 10^−5^ mol/L, which is more sensitive than those reported by current detection methods such as Phenol spectrophotometry (Lin and Ye, 2010) (LOD is nearly 8.84 mol/L) or gas chromatography (Walsh, 2001) (LOD is nearly 3.45 × 10^−3^ mol/L). However, there are also some problems and limitations in our studies. The method needs the corresponding apparatus to accomplish the detection which is same as the other methods. Further development of the method may provide a more convenient and effective detection for nitrite in complex samples.

## Data Availability Statement

All datasets generated for this study are included in the article/supplementary material.

## Author Contributions

HL, ZP, and YZ: methodology, formal analysis, investigation, data curation, and writing—original draft. XL and XZ: conceptualization, validation, writing—review and editing, supervision, project administration, and funding acquisition. SX, MS, and LJ: resources, writing—original draft.

## Conflict of Interest

The authors declare that the research was conducted in the absence of any commercial or financial relationships that could be construed as a potential conflict of interest.
